# Low vision aids and age are associated with Müller-Lyer illusion in congenital visually impaired children

**DOI:** 10.3389/fpsyg.2023.1278554

**Published:** 2023-11-24

**Authors:** Na Lin, Bichi Chen, Maoyuan Yang, Fan Lu, Ruzhi Deng

**Affiliations:** ^1^National Clinical Research Center for Ocular Diseases, Eye Hospital, Wenzhou Medical University, Wenzhou, China; ^2^School of Ophthalmology and Optometry, Wenzhou Medical University, Wenzhou, China

**Keywords:** Müller-Lyer illusion, visual impairment, age, low vision aids, developmental trend

## Abstract

**Background:**

The correlation between visual impairment and Müller-Lyer illusion is not yet elucidated. This study aimed to explore the connection between visual status, age, and the intensity of Müller-Lyer illusion in congenitally visually impaired and visually healthy children aged 4–17 years. Additionally, the developmental trends were compared.

**Methods:**

This cross-sectional study included 125 visually impaired children (age: 10.59 ± 4.05 years), among them, 53 had utilized low vision aids (LVAs) and 133 healthy controls (age: 11.33 ± 3.39 years). The participants were presented with Müller-Lyer illusion stimuli via binocular and engaged in a two-alternative forced choice task to quantify the illusion intensity. Pertinent factors including age, gender, residence, binocular distant best-corrected visual acuity and LVAs usage history, were assessed.

**Results:**

The visually impaired group exhibited significantly elevated illusion intensity compared to the healthy group (9.74 ± 2.89% vs. 5.42 ± 3.81%, *p* < 0.001), and visually impaired participants who had used LVAs exhibited significantly lower intensity compared to those had not (9.13 ± 3.00% vs. 10.19 ± 2.74%, *p* = 0.043). Multivariate generalized estimation equations revealed that visual impairment [odds ratio (OR) = 2.75, *p* < 0.001] and age (OR = 0.60, *p* < 0.001) were associated with illusion intensity in all participants, while history of LVAs usage (OR = 0.49, *p* = 0.045) and age (OR = 0.61, *p* < 0.001) were negatively correlated in visually impaired group. A significantly negative correlation was found between illusion intensity and age 4–17 years in the subgroups of visually impaired who had used LVAs (*Y* = −0.54X + 15.06, *R*^2^ = 0.56), who had not used (*Y* = −0.49X + 15.24, *R*^2^ = 0.51), and healthy controls (*Y* = −0.50X + 11.18, *R*^2^ = 0.21); all *p*-values were < 0.001.

**Conclusion:**

Children aged 4–17 years afflicted with congenital visual impairment exhibited a heightened intensity of Müller-Lyer illusion compared to visually normal counterparts, LVAs usage experience could reduce this higher intensity. The developmental trajectory of illusion intensity declined consistently with age across all three groups. The abnormal visual experiences during early-life may adversely affect integration in congenitally visually impaired children, and LVAs could facilitate this functional development.

## 1 Introduction

Visual integration constitutes a fundamental facet of natural perception, underpinning the capacity for learning and communication within individuals (Ionta, 2021). The mechanism of visual perception underlies the apprehension of the surrounding environment and is intrinsically linked to the generation of perceptual illusions (Eagleman, 2001). Among these illusions, the Müller-Lyer phenomenon is as a well-established geometric distortion, wherein the presentation of two parallel lines of identical length results in the erroneous perception of the line adorned with outward-pointing arrowheads (< >) as shorter, while the line featuring inward-pointing arrowheads (> <) is often misconceived as longer (Müller-Lyer, 1889). This effect is so profound that even after the complete disclosure of its nature, participants continue to report divergent line lengths (Pylyshyn, 1999). Thus, Müller-Lyer illusion served as a potent and non-invasive instrument that offers insights into the intricate terrain of human perceptual processing and cerebral mechanisms (Bruno et al., 2008; Mancini et al., 2011b; Gandhi et al., 2015).

Despite its status as a visual illusion, the correction between visual impairment and the Müller-Lyer illusion is controversial. Zhang et al. (2017) revealed that military personnel exhibited diminished Müller-Lyer intensity compared to university students of equivalent age, thereby lending credence to experiential interpretation. Additional studies have also indicated the significant influence of visual experience on illusion perception (Gillam, 1980; Howe and Purves, 2005). Nevertheless, recent evidence has suggested that Müller-Lyer illusion manifests as an inherent phenomenon unaffected by an individual’ s previous visual encounters (Gandhi et al., 2015). In the case of children and adults afflicted with visual impairment and restricted visual exposure, the deployment of low vision aids (LVAs) has emerged as a prevalent clinical strategy to ameliorate the impairment (Margrain, 1999; Virgili et al., 2018). Furthermore, the developmental trajectory of the Müller-Lyer illusion in children with congenital visual impairment is yet to be elucidated.

The current study aimed to scrutinize the nexus between Müller-Lyer illusion intensity, visual status, and age (4–17 years), encompassing those with and without congenital visual impairment. Additionally, we conducted a comparative analysis of the developmental trends in illusion intensity across three groups: visually impaired participants had utilized LVAs, visually impaired participants had no experience of using such aids, and visually healthy participants.

## 2 Methods

### 2.1 Study design

This cross-sectional study was conducted between June 2020 and October 2022 at The Eye Hospital of Wenzhou Medical University, Zhejiang, China.

The inclusion criteria were as follows: (1) < 18 years-old; (2) diagnosed as simple congenital refractive system diseases or visual health. Visual impairment was diagnosed in accordance with the 2019 criteria established by the World Health Organization. In this context, visual impairment encompassed cases wherein binocular near and distant presenting visual acuity (PVA) and best-corrected visual acuity (BCVA) exceeded 0.5 logarithm of the minimum angle of resolution (logMAR). Conversely, visual health was defined by near and distant PVA not surpassing 0.2 logMAR; (3) able to comprehend the test material and communicate effectively (we conducted simple tests, such as comparison of the length of two line segments); (4) normal eye movement, allowing successful engagement with graphic stimuli on the screen; (5) fundus photography or optical coherence tomography tests showed no abnormality in fundus structure; (6) right-handed.

The exclusion criteria were as follows: (1) intermittent strabismus and tropia; (2) the disparity in BCVA between both eyes remained under 0.1 logMAR; (3) have a history of neurological diseases, such as hydrocephalus, epilepsy, intracranial tumors, peripheral neuritis, leukodystrophy, and spinocerebellar ataxia.

Two certified ophthalmologists from the Department of Pediatric Ophthalmology evaluated the parameters, including binocular distant PVA and BCVA (utilizing the tumbling E early treatment diabetic retinopathy study chart, Good-Lite Vision, Houston, USA), binocular near PVA and BCVA (using the near tumbling E logarithms visual acuity chart, Xingkang Medical Technology, Wenzhou, China), stereoacuity (using the Randot Stereotest, Stereo Optical Company, Chicago, IL, USA), eye alignment (assessed via the cover-uncover test and alternate prism cover test), and eye movement in the participants.

The ethics approval for this study was obtained from the Institutional Ethics Committee of the Eye Hospital of Wenzhou Medical University (2020-075-K-67-01 and 2020-075-K-67-02), with strict adherence to the principles outlined in the Declaration of Helsinki. An informed consent was obtained from all participating children and their legal guardians. The fingerprint of the right index finger for signature was considered as consent in young children with the help of their legal guardians.

### 2.2 Participants

A total of 125 subjects presenting with congenital visual impairment [59 females; mean age: 10.59 ± 4.05 (age range: 4.05–17.99) years; 70 residing in rural areas; 53 had utilized LVAs; Table 1] and 133 visually healthy controls [59 females; mean age: 11.33 ± 3.39 (age range: 4.30–17.92) years; 66 residing in rural areas; Table 1] were enrolled in this study.

**Table 1:** Characteristics of visual impairment and healthy participants.

Characteristics	Results			
	Visual impairment group (*n* = 125)	Visual healthy group (*n* = 133)	t/Z/χ^2^ value	*p* value
Intensity of M-L illusion, %	9.74 ± 2.89 (0.50–18.00)	5.42 ± 3.81 (0.50–14.00)	11.84^a^	0.001
Age, years	10.59 ± 4.05 (4.05–17.99)	11.33 ± 3.39 (4.30–17.92)	8.17^a^	0.113
Gender, *n* (%)			0.21^b^	0.647
Female	59 (47.20%)	59 (44.40%)		
Male	66 (52.80%)	74 (55.60%)		
Residence status, *n* (%)			0.19^b^	0.673
Rural area	70 (56.00%)	71 (53.40%)		
Urban area	55 (44.00%)	62 (46.60%)		
Binocular distant BCVA, logMAR	0.70 (0.60–0.92) (0.52–1.10)	0.10 (0.05–0.10) (−0.08–0.10)	–14.33^c^	<0.001
0.70–0.52	57 (45.60%)	–		
1.10–0.70	68 (54.40%)	–		
≤0.00	–	33 (24.90%)		
0.10	–	100 (75.10%)		
Binocular near BCVA, logMAR	0.70 (0.60–0.92) (0.52–1.22)	0.10 (0.10–0.10) (−0.08–0.10)	−14.44 ^c^	<0.001
0.70–0.52	59 (47.20%)	–		
1.30–0.70	66 (52.80%)	–		
≤0.00	–	24 (19.50%)		
0.10	–	107 (80.50%)		
History of LVAs usage			–	–
No	72 (57.6%)	–		
Yes	53 (42.4%)	–		

M-L, Müller-Lyer illusion; BCVA, Best-Corrected Visual Acuity; logMAR, Logarithm of the Minimum Angle of Resolution; LVAs, Low Vision Aids.

^a^Independent *T*-Test; ^b^Person Chi-Square Test; ^c^Mann–Whitney Rank-Sum Test.

### 2.3 Measurements

#### 2.3.1 Stimuli

**Figure 1:**
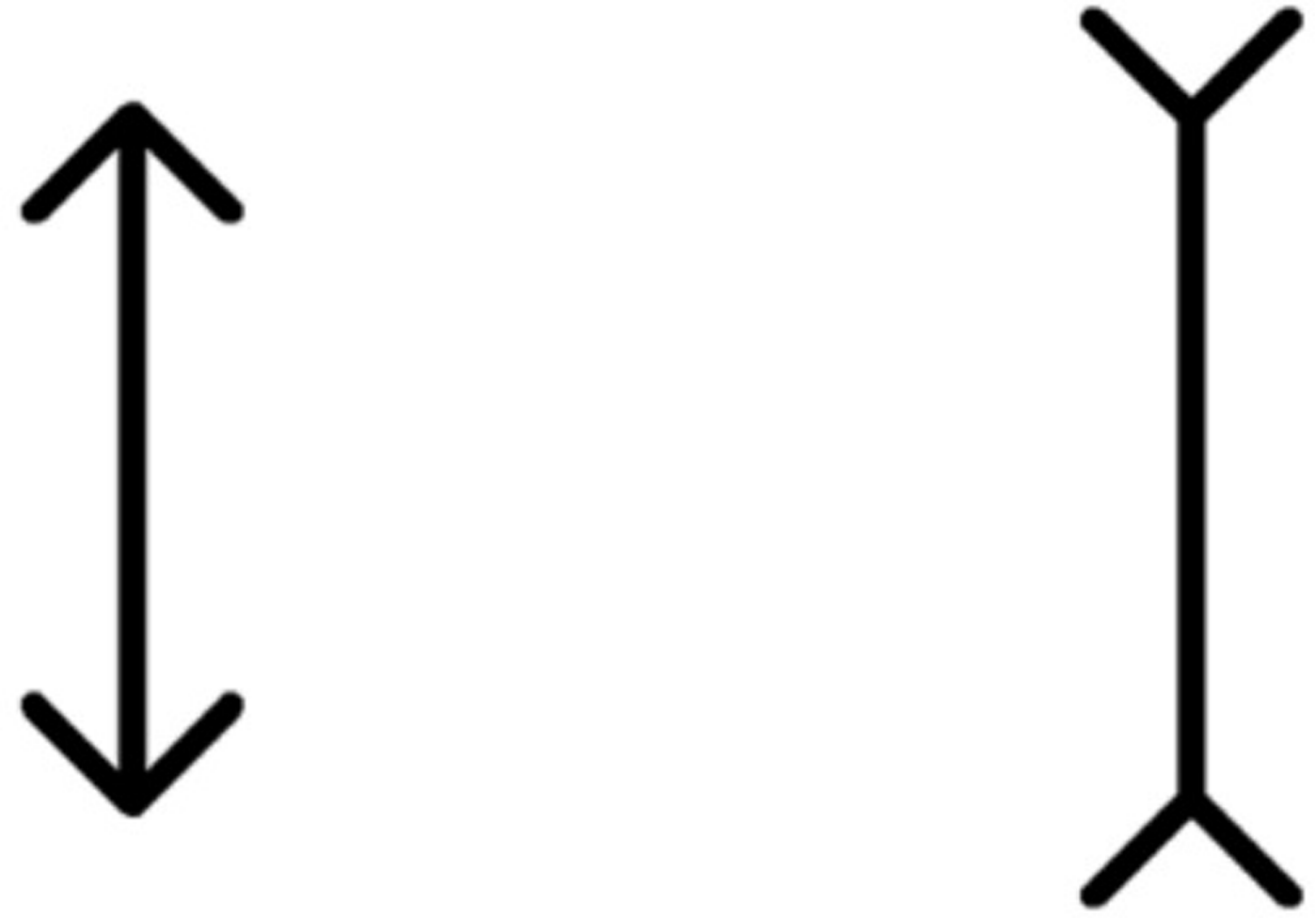
Müller-Lyer illusion stimulus pattern. The standard line, measuring 250 pixels in length and 12.5 pixels in width, is juxtaposed against the arrow stimulus spanning 50 pixels. This illustration is observed on a screen projecting a resolution of 1,366 × 768 pixels, with the angle between the two arrows set at 90°.

The classic Müller-Lyer illusion pattern served as the stimulus, displayed in black against a white screen with a 100% contrast (Figure 1). The standard line (featuring an outward-pointing arrowhead) measured 250 pixels in length and 12.5 pixels in width (equal to visual angles of 1.5° and 0.075°, respectively, when tested at 40 cm; these angles corresponded to visual acuity levels of 2.0 logMAR and 0.7 logMAR). The arrow stimulus occupied 50 pixels on the screen, with a 90° angle between the two arrows.

#### 2.3.2 Apparatus and procedure

The image stimuli were presented on a 14-inch laptop screen (ThinkPad Edge E431, Lenovo Corporation, Beijing, China) with a screen resolution of 1,366 × 768 pixels. A C# script (Visual Studio, version 25.0, Microsoft, USA) facilitated image display and response recording process.

We develpoed a program based on the adjustment method (Manning et al., 2017) to measure the illusion threshold. In this two-alternative forced choice task, participants were tasked with selecting the longer line segment by pressing the key (left/right) on a touch screen or mouse button when viewed binocularly. The program iteratively altered the length of the comparison stimulus to converge with the standard stimulus, contingent on the participants’ choices. An enhanced variable step size least mean square adaptive algorithm (Jiao et al., 2013) was implemented to regulate the length adjustments. Each threshold data point entailed a minimum of 20 trials when the standard deviation of the most recent five trials fell below 0.7 times the minimum length step size (McKelvie, 1984; Plesch et al., 2019). The threshold data were computed as the average value derived from the last five lengths of the comparison stimulus.

The test program did not maintain fixed left and right positions for the comparison and standard stimul to minimize positional bias. A minimum of two valid data points was collected during each session (validity criteria: the difference between the two data points was <5%), with the average value constituting the final measurement result. The intensity of the Müller-Lyer illusion was defined as the absolute difference between the illusion threshold and 100%, in the unit of %. Notably, a substantial disparity between the illusion threshold and 100% indicated a robust Müller-Lyer illusion effect, thus yielding a heightened illusion intensity in the task.

Preceding the testing phase, two trained postgraduates elucidated the task to the participants. Then, participants were required to spontaneously select the longer line segment within this two-alternative forced choice task. Throughout the test, the experimenters refrained from providing feedback on the correctness of the participants’ choices but encouraged them to complete the test. The tests were conducted in a well-lit and quiet room. Guardians refrained from offering verbal or behavioral prompts but could encourage them to complete the test. Also, we encouraged the participating children to measure again or the next day in the event of failure.

#### 2.3.3 Repeatability test results of the program

A total of 15 children afflicted with visual impairment [7 females; mean age: 8.42 ± 4.41 (range: 4.10–17.73) years], fulfilling the stipulated inclusion and exclusion criteria mentioned aboved underwent intensity measurements three times via the self-developed program. The intragroup correlation coefficients for Müller-Lyer illusion intensity were computed as 0.953, 0.949, and 0.954, respectively (all *p*-values <0.001), and repetition coefficient percentages were recorded as 3.6, 4.0, and 3.8%, respectively. A Bland–Altman plot depicted narrow 95% limits of agreement for repeated measurements, devoid of significant deviations. The average trials per dataset was 32.3 ± 6.4, with a mean time of 2.8 ± 0.6 min.

### 2.4 Statistical analysis

Continuous variables were presented as mean ± standard deviation or median (interquartile range, IQR) where appropriate. Normal distribution variables were analyzed through independent *t*-tests, one-way repeated measures analysis of variance, or the least significant difference test. Non-normally distributed variables were subjected to the chi-square test and Mann–Whitney rank- sum test. We used univariate Generalized Estimation Equation (GEE) analysis to explore the potential factors related to the intensity of Müller-Lyer illusion, and then put factors with *p*-values <0.05 into multivariate GEE analysis for adjusting the potential danger of co-variation between variables. We employed partial correlation analysis to delineate the correlation between Müller-Lyer illusion intensity and visual acuity when age was controlled to eliminate its influence and that between illusion intensity and age when visual acuity was controlled. We employed the linear regression equation to elucidate the correlation between age and illusion intensity and the general linear model’s covariance analysis to assess the change rate differences.

Notably, the Spearman’s rank correlation coefficients between distant and near binocular BCVA in all participants, visual impairment group and visually healthy controls were 0.971, 0.785, and 0.902, respectively (all *p*-values <0.001), prompting the factor analysis to focus solely on binocular distant BCVA. The diverse vision loss causes of the congenitally visually impaired participants precluded uniform consistency between both eyes, prompting separate counts. For eyes with multiple diagnoses, only the primary diagnosis was considered in the analysis.

Data were subjected to double-blind entry using Epidata (version 3.1.2701.2008, China), while SPSS statistical software (version 21.0, SPSS, Inc., Chicago, IL, USA) facilitated the data analysis process. All *p*-values were calculated as two-sided, with statistical significance established at <0.05.

## 3 Results

### 3.1 Demographics of participants

The visual impairment group exhibited significantly elevated Müller-Lyer illusion intensity compared to the visually healthy group (9.74 ± 2.89% vs. 5.42 ± 3.81%, *p* < 0.001). Nonetheless, no significant differences were observed in terms of gender, residence, and age distribution between the two groups (all *p*-values >0.05, Table 1).

The etiological factors contributing to congenital visual impairment encompassed congenital cataracts (48.80%, 122 eyes), ametropic amblyopia (39.20%, 98 eyes), congenital corneal leukoplakia (8.00%, 20 eyes), and microspherophakia (4.00%, 10 eyes). In the visually impaired group, participants who had used LVAs exhibited lower Müller-Lyer illusion intensity compared to those had not used such aids (9.13 ± 3.00% vs. 10.19 ± 2.74%, *p* = 0.043), while no significant discrepancies were observed in terms of age, binocular distant and near BCVA between the two subgroups (Table 2).

**Table 2:** Characteristics of visual impairment participants (*n* = 125).

Characteristics	Results			
	Had used LVAs (*n* = 53)	Had not used LVAs (*n* = 72)	*t*/*Z*/*χ*^2^ value	*p* value
Intensity of M-L illusion, %	9.13 ± 3.00 (5.00–15.17)	10.19 ± 2.74 (5.00–18.00)	−2.05^a^	0.043
Age, years	10.98 ± 4.17 (4.05–17.99)	10.21 ± 3.96 (4.20–17.90)	0.92^a^	0.359
Gender, *n* (%)			3.27^b^	0.071
Male	23 (43.40%)	43 (59.70%)		
Female	30 (56.60%)	29 (40.30%)		
Residence status, *n* (%)			0.01^b^	0.907
Urban area	23 (43.40%)	32 (44.40%)		
Rural area	30 (56.60%)	40 (55.60%)		
Binocular distant BCVA, logMAR	0.70 (0.70–0.92) (0.52–1.22)	0.70 (0.70–0.92) (0.52–1.22)	−0.22^c^	0.829
0.70–0.52	24 (45.30%)	33 (37.10%)		
1.10–0.70	29 (54.70%)	39 (62.90%)		
Binocular near BCVA, logMAR	0.70 (0.70–0.92) (0.52–1.22)	0.70 (0.70–0.92) (0.52–1.22)	−0.17 ^c^	0.866
0.70–0.52	24 (45.30%)	33 (37.10%)		
1.30–0.70	29 (54.70%)	39 (62.90%)		

M-L, Müller-Lyer illusion; LVAs, Low Vision Aids; BCVA, Best-Corrected Visual Acuity; logMAR, Logarithm of the Minimum Angle of Resolution.

^a^Independent T-Test; ^b^Person Chi-Square Test; ^c^Mann–Whitney Rank-Sum Test.

### 3.2 Factors related to the intensity of Müller-Lyer illusion

Univariate GEE analysis showed that visual impairment [*β* = 1.32, odds ratio (OR) = 3.74, 95% confidence interval (CI): 1.82–4.34, *p* < 0.001] and age (*β* = −0.57, OR = 0.57, 95% CI: 0.51–0.63, *p* < 0.001) were significantly related to illusion intensity in all visually impaired and healthy participants. These two variables were then entered into multivariate GEE analysis, which highlighted the relevance of visual impairment (*β* = 1.01, OR = 2.75, 95% CI: 1.75–3.97, *p* < 0.001) and age (*β* = −0.52, OR = 0.60, 95% CI: 0.55–0.65, *p* < 0.001) with respect to illusion intensity (Table 3).

**Table 3:** Factors potentially related to the intensity of Müller-Lyer illusion in visual impairment and healthy participants (*n* = 258).

Factors	Univariate				Multivariate			
	*β*	OR	95%CI	*p* value	*β*	Adjusted OR	95%CI	*p* value
Age, years	−0.57	0.57	0.51–0.63	**<0.001**	−0.52	0.60	0.55–0.65	**<0.001**
Gender								
Female	Reference				–	–	–	–
Male	0.33	1.40	0.52–3.74	0.507	–	–	–	–
Residence status								
Rural area	Reference				–	–	-	
Urban area	0.11	1.12	0.42–3.01	0.820	–	–	–	–
Visual impairment								
No	Reference				Reference			
Yes	1.32	3.74	1.82–4.34	**<0.001**	1.01	2.75	1.75–3.97	**<0.001**

OR, Odds Ratio; CI, Confidence Interval.

OR with statistical significance is listed in bold.

In the visual impairment subgroup, univariate GEE analysis showed that age (*β* = −0.52, OR = 0.60, 95% CI: 0.55–0.65, *p* < 0.001), binocular distant BCVA between 1.10 and 0.70 logMAR (*β* = −1.33, OR = 0.26, 95% CI: 0.10–0.71, *p* = 0.009), and the history of LVAs usage (*β* = −1.06, OR = 0.35, 95% CI: 0.13–0.96, *p* = 0.041) were significantly negatively related to illusion intensity. These three variables were input into multivariate GEE analysis, which indicated a significantly inverse correlation between illusion intensity and the history of LVAs usage (*β* = −0.72, OR = 0.49, 95% CI: 0.24–0.98, *p* = 0.045), as well as age (*β* = −0.50, OR = 0.61, 95% CI: 0.55–0.66, *p* < 0.001). Following adjustment, no significant association was observed between illusion intensity and binocular distant BCVA level (Table 4).

**Table 4:** Factors potentially related to the intensity of Müller-Lyer illusion in visual impairment participants (*n* = 125).

Factors	Univariate				Multivariate			
	*β*	OR	95%CI	*p* value	*β*	Adjusted OR	95%CI	*p* value
Age, years	−0.52	0.60	0.55–0.65	**<0.001**	−0.50	0.61	0.55–0.66	**<0.001**
Gender								
Female	Reference				–	–	–	–
Male	0.90	2.46	0.90–6.72	0.080	–	–	–	–
Residence status								
Rural area	Reference				–	–	–	–
Urban area	−0.17	0.85	0.30–2.35	0.747	–	–	–	–
Binocular distant BCVA, logMAR								
0.70–0.52	Reference					Reference		
1.10–0.70	−1.33	0.26	0.10–0.71	**0.009**	−0.40	0.67	0.34–1.33	0.256
History of LVAs usage								
No	Reference				Reference			
Yes	−1.06	0.35	0.13–0.96	**0.041**	−0.72	0.49	0.24–0.98	**0.045**

OR, Odds Ratio; CI, Confidence Interval; BCVA, Best-Corrected Visual Acuity; logMAR, Logarithm of the Minimum Angle of Resolution; LVAs, Low Vision Aids.

OR with statistical significance is listed in bold.

### 3.3 Correlation between the intensity of Müller-Lyer illusion and binocular distant BCVA

**Figure 2:**
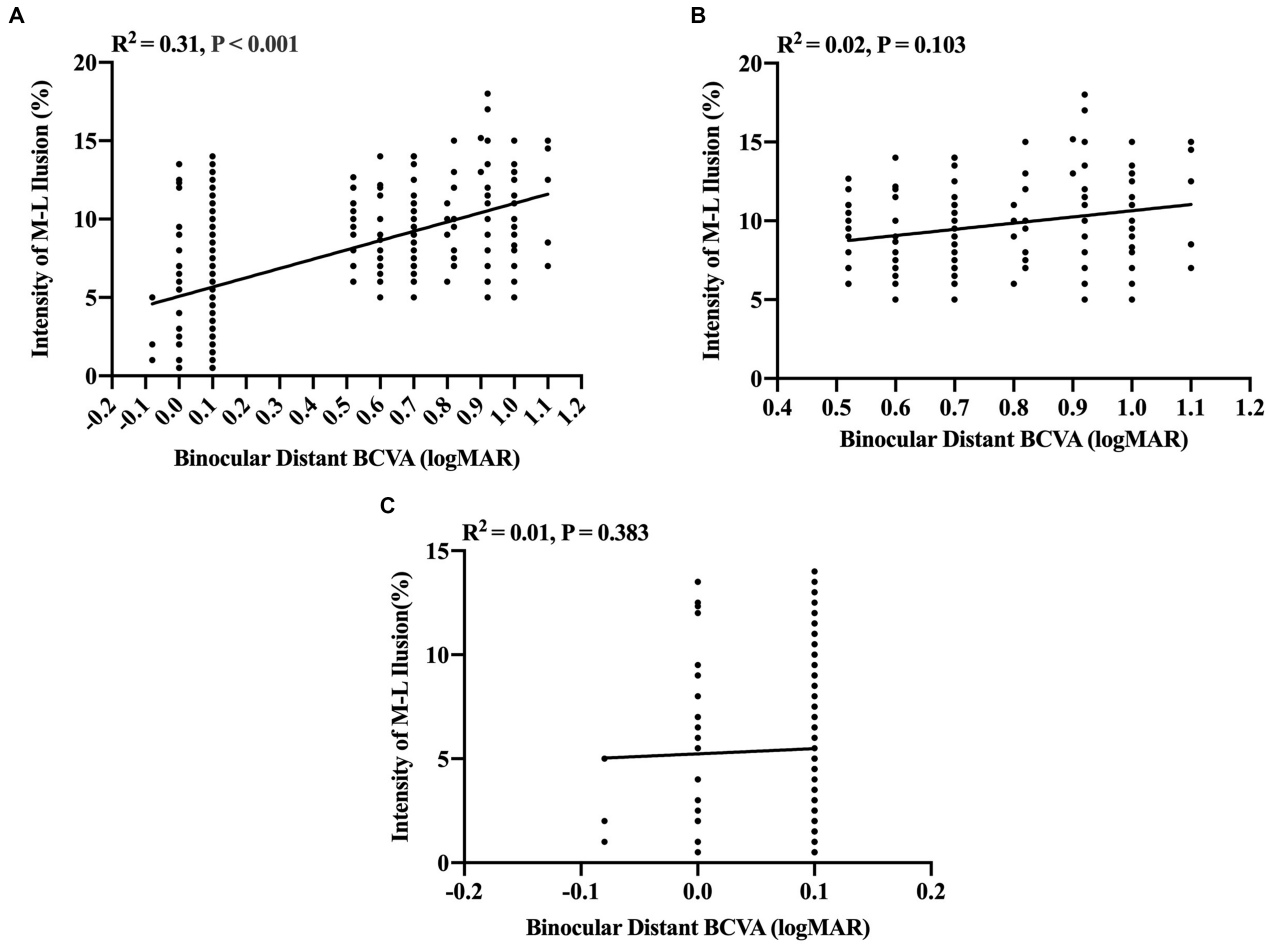
Partial correlation analysis between binocular distant BCVA (logMAR) and intensity of M-L illusion (%). **(A)** A significant positive correlation between binocular distant BCVA and Müller-Lyer illusion intensity for all participants (*n* = 258). **(B)** The correlation between Müller-Lyer illusion intensity and binocular distant BCVA in the congenitally visually impaired group (*n* = 133) when binocular distant BCVA ≥0.52 logMAR, test power was 0.9999. **(C)** The correlation between Müller-Lyer illusion intensity and binocular distant BCVA in the visually healthy group (*n* = 125) when binocular distant BCVA ≤0.10 logMAR, test power was 0.9999. The X-axis denotes the Müller-Lyer illusion intensity values (%), while the Y-axis represents the values of binocular distant BCVA (logMAR). The regression lines are derived from linear regression analysis, depicting the extent of the linear correlation. The amount of variance accounted for by this linear correlation is indicated by the coefficient of determination (R^2^). M-L, Müller-Lyer illusion; BCVA, Best-Corrected Visual Acuity; logMAR, Logarithm of the Minimum Angle of Resolution.

Further insights into the linear correlation between illusion intensity and visual acuity were obtained through partial correlation analysis. These findings disclosed a significantly positive correlation between binocular distant BCVA between −0.10 and 1.10 logMAR and illusion intensity (*R*^2^ = 0.31, *p* < 0.001, Figure 2A). However, no significant correlations were discerned between binocular distant BCVA and illusion intensity in either the visual impairment group when the binocular distant BCVA was between 0.52 and 1.10 logMAR (*R*^2^ = 0.02, *p* = 0.103, test power was 0.9999, Figure 2B) or the visually healthy group when the binocular distant BCVA was between −0.10 and 0.10 logMAR (*R*^2^ = 0.01, *p* = 0.383, test power was 0.9999, Figure 2C).

### 3.4 Correlation between the intensity of Müller-Lyer illusion and age in the three groups

**Figure 3:**
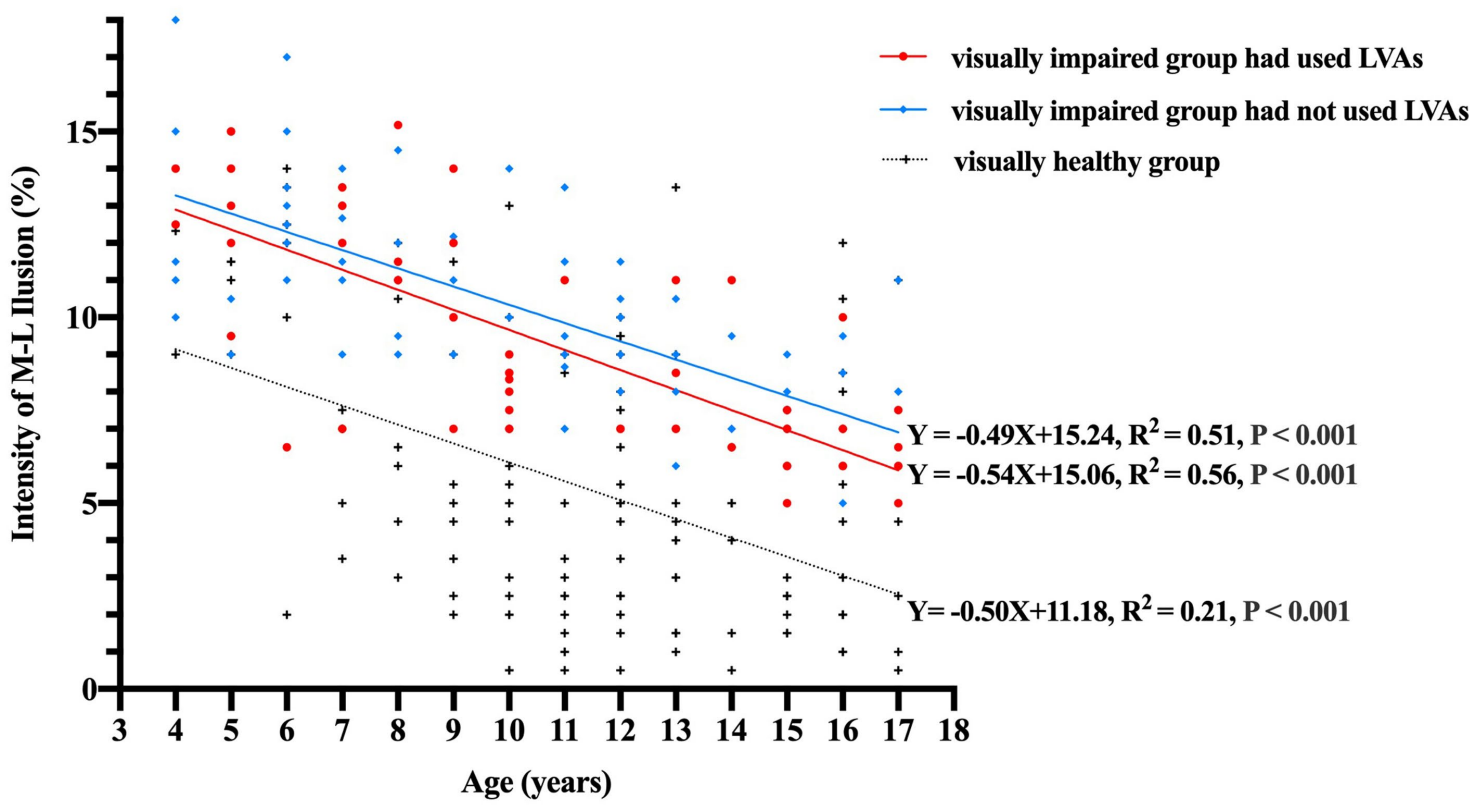
Correlation between the intensity of M-L illusion (%) and age (years) among the three groups. The data points are represented by red circles for visually impaired participants who had utilized LVAs (*n* = 53), blue diamonds for visually impaired participants who had not utilized LVAs (*n* = 72), and black crosses for visually healthy controls (*n* = 133). The linear regression equations for each group signify that illusion magnitudes experience a notable decline with increasing age (all *p*-values <0.001). Moreover, the covariance analysis utilizing a general linear model demonstrates that the change rates between each pair of groups are not statistically significant (change rate − 0.54 vs. −0.49, *F* = 0.31, *p* = 0.579; change rate − 0.49 vs. −0.50, *F* = 0.02, *p* = 0.881; change rate − 0.54 vs. −0.50, *F* = 0.06, *p* = 0.810). M-L, Müller-Lyer illusion; LVAs, Low Vision Aids.

When accounting for binocular distant BCVA as a controlling factor to eliminate its influence, the partial correlation coefficients between illusion intensity and age 4–17 years in the subgroups of visually impaired participants who had used LVAs, visually impaired participants who had not used LVAs, and visually healthy participants were found to be −0.75, −0.70, and − 0.46, respectively (all *p*-values <0.001). The linear regression equations established for these subgroups were as follows: *Y* = −0.54X + 15.06 (*R*^2^ = 0.56), *Y* = −0.49X + 15.24 (*R*^2^ = 0.51), and *Y* = −0.50X + 11.18 (*R*^2^ = 0.21), respectively (all *p*-values <0.001, Figure 3). The general linear model’s covariance analysis revealed that the change rates between any two groups were not significant (change rate − 0.54 vs. –0.49, *F* = 0.31, *p* = 0.579; change rate − 0.49 vs. –0.50, *F* = 0.02, *p* = 0.881; change rate − 0.54 vs. –0.50, *F* = 0.06, *p* = 0.810).

## 4 Discussion

The present study demonstrated that children with congenital visual impairment exhibit heightened intensity of the Müller-Lyer illusion compared to their visually healthy counterparts. Moreover, this augmented intensity is inversely linked to a history of employing LVAs and advancing age. The observed trajectory of illusion intensity across the three groups diminishes with advancing age. These findings substantiated that the Müller-Lyer illusion manifests with an individual’s accrued visual experience and cognitive maturation. Notably, the use of LVAs appears to foster this functional development among individuals who have congenital visual impairment. To the best of our knowledge, this is the first quantitative study that elucidates the deficits in Müller-Lyer illusion integration among young children grappling with congenital visual impairment.

Our findings substantiated the foremost impact of visual impairment (OR = 2.75) on Müller-Lyer illusion intensity in children aged 4–17 years. This observation aligns with Keep’s assertion (Keep et al., 2018) regarding the plausible pivotal role of visual acuity in this perceptual phenomenon demonstrated in dogs. Similarly, Narinesingh et al. (2014, 2015) have accentuated that children and adults afflicted with amblyopia exhibit diminished susceptibility to McGurk effect—an analogous visual geometric illusion. Their findings imply that early-life visual deprivation can potentially trigger aberrations in real-world perceptual processing. The reduced visual exposure experienced by children with congenital visual impairment during crucial phases of visual development may hinder their cognitive and personality growth (Pasqualotto and Proulx, 2012). Notably, these limitations in an individual may have an adverse effect on cognitive proficiency and logical reasoning capabilities (Levelt and Hübener, 2012). However, Gandhi et al. (2015) presented opposite results. Their study highlighted that children with severe congenital cataracts who gained vision post-surgery could promptly perceive the Müller-Lyer illusion, challenging the theory that prior visual experience is a prerequisite for this phenomenon. Nonetheless, although severely visually impaired, the children in the study did not meet the criteria of complete blindness, as their visual acuity ranged from hand motion to counting fingers, they had the opportunity to acquire visual experience prior to surgical intervention. Additionally, the qualitative measurement of Müller-Lyer illusion interferes with the interpretation of the findings. Interestingly, our study deviates from the anticipated trend, revealing that in the visually impaired group, poor visual acuity does not correspond to heightened Müller-Lyer illusion intensity. This discrepancy could potentially be attributed to the concept that visual experience is not solely restricted to visual input, rather encompasses an array of sensory modalities, including tactile and auditory senses (Kupers et al., 2010). Auditory information can engage the visual cortex through direct pathways linking the primary auditory cortex to visual areas (Falchier et al., 2002) or via indirect relay connections involving multimodal associative cortices (Falchier et al., 2010).

The impaired visual integration in our study adds to the growing body of evidence that visual impairment is associated with low-level deficits, such as visual acuity, and an array of deficits in higher-level perceptual processes. Animal studies have shown that experimentally induced low vision acuity is associated with abnormalities in the architecture and functional properties of the primary visual cortex (V1) (Wiesel, 1982; Fregnac and Imbert, 1984). The studies in visually impaired humans have also suggested complex perceptual deficits in downstream extrastriate and later specialized cortical areas (Simmers and Bex, 2004; Simmers et al., 2005). The finding that children with congenital visual impairment show persistent deficits in visual integration approaching adulthood, which differentiates it from other conditions, such as schizophrenia or autism, that only delay the development of the integration rather than causing permanent deficits (Pearl et al., 2009; Stevenson et al., 2014; Choi et al., 2023). This phenomenon may be explained by the difference in the pathophysiology underlying these diverse conditions. Schizophrenia and autism involve neural abnormalities, impaired integration of inhibitory and excitatory neural responses, and impaired top-down feedback mechanisms that may adversely affect the performance of visual illusion tasks (Park et al., 2022; Zhu et al., 2023). Conversely, the bottom-up effects of congenital low vision on visual integration during the critical development period are observed because the spatiotemporal deficits begin early in area V1.

The current study observed a marked decrease in Müller-Lyer illusion susceptibility across the age range of 4–17 years, spanning both visually impaired and healthy children. Binet (1895) postulated that this trend could be attributed to the maturation of cognitive faculties with the growth of children. Over time, they acquire the capacity to rectify their perceptual discrepancies and consequently attenuate the manifestation of the illusions. The interpretation of line length disparities in the context of Müller-Lyer illusion has been associated with cumulative exposure to the tridimensional world (Gillam, 1980; Howe and Purves, 2005). Functional magnetic resonance imaging of the brain has illuminated the intricate neural underpinnings of the Müller-Lyer illusion, implicating the lateral occipital cortex and the upper parietal cortex along the ventral and dorsal pathways (Weidner and Fink, 2007), as well as interactions within the visual and frontal parietal cortices (Mancini et al., 2011a; Zhang et al., 2013). The cognitive capacities evolve with age, typically reaching adult levels at approximately 10–12-years-old (Chelune and Baer, 1986; Rosselli and Ardila, 1993); thus, it can be surmised that the genesis of the Müller-Lyer illusion is intertwined with visual experience and cognitive maturation.

Interestingly, we observed that the application of LVAs reduces the intensity of Müller-Lyer illusion. This finding, coupled with the absence of correlation between illusion intensity and visual acuity among congenitally visually impaired children, lends credence to the theory that early-life abnormal visual experiences can induce deviations in real-world perceptual processing that transcend mere visual acuity (Narinesingh et al., 2014). The fundamental aim of LVAs is to empower individuals with visual impairment to engage in daily tasks reliant on visual cues seamlessly (Virgili et al., 2018). This, in turn, fosters an accumulation of visual experience. Notably, the global number of moderate and severe visually impaired cases in 2020 was about 295 million and might rise to 474 million in 2050 (GBD 2019 Blindness and Vision Impairment Collaborators, and Vision Loss Expert Group of the Global Burden of Disease Study, 2021). However, the adoption of visual impairment rehabilitation services and the utilization of LVAs is limited (Mwilambwe et al., 2009). Our study echoes this trend, with a mere 42.4% of visually impaired participants utilizing LVAs, thereby emphasizing the urgency to bolster the outreach and implementation of visual impairment rehabilitation services and to encourage the widespread adoption of LVAs.

The impact of residence on Müller-Lyer illusion intensity in this study was negligible, deviating from previous conclusions. For instance, Segall et al. (1963) discovered that children residing in rural areas exhibited higher illusion intensity compared to their urban counterparts. This variance was attributed to rural and grassland-dwelling children with broad visual exposure to their environment, resulting in reduced geometric line stimuli and limited comprehension of intricate figures. On the other hand, simple geometric illusions, such as the Müller-Lyer illusion, were prevalent in educated, industrialized, and affluent democratic cultures (Henrich et al., 2010). However, most of the studies were conducted before 2005, and the living environments in rural areas of China have converged with urban settings in the past decade.

Nevertheless, the present study has some limitations. Herein, we focused exclusively on children with congenital visual impairment, omitting middle-aged and elderly adults with the same condition. Such individuals often receive treatment and are less likely to sustain prolonged periods of blurred vision due to increased medical awareness and widespread medical screenings. Moreover, the four types of primary diagnosed diseases of participants with visual impairments are heterogeneous, and the inclusion numbers are limited owing to the various factors that might contribute to the development of illusion. Additionally, the effect of types and duration of LVAs on the Müller-Lyer illusion has not yet been analyzed. In subsequent studies, we will continue to expand the sample size and explore the impact of types of diseases and LVAs on Müller-Lyer illusion.

In conclusion, the current study corroborates a correlation between Müller-Lyer illusion and visual impairment with respect to age. Children aged 4–17 years afflicted with congenital visual impairment exhibited a heightened intensity of Müller-Lyer illusion compared to visually normal counterparts. Notably, this high intensity is reduced with the history of LVAs usage and advancing age. Furthermore, a consistent decline was noted in illusion intensity across all three groups in an age-dependent manner. We also analyzed the detrimental impact of abnormal visual experiences in early-life on visual integration among children with congenital visual impairment while highlighting the potential of LVAs in fostering functional development.

## Data availability statement

The original contributions presented in the study are included in the article/supplementary material, further inquiries can be directed to the corresponding authors.

## Ethics statement

This study garnered approval from the institutional ethics committee of the Eye Hospital of Wenzhou Medical University (2020-075-K-67-01 and 2020-075-K-67-02). The studies were conducted in accordance with the local legislation and institutional requirements. Written informed consent for participation in this study was provided by the participants’ legal guardians/next of kin.

## Author contributions

NL: Data curation, Formal analysis, Funding acquisition, Project administration, Writing – original draft. BC: Data curation, Formal analysis, Project administration, Validation, Writing – original draft. MY: Data curation, Formal analysis, Validation, Writing – original draft. FL: Funding acquisition, Methodology, Supervision, Writing – review & editing, Project administration. RD: Funding acquisition, Methodology, Project administration, Writing – review & editing, Supervision.
